# Investigation of mutations in the *HBB* gene using the 1,000 genomes database

**DOI:** 10.1371/journal.pone.0174637

**Published:** 2017-04-05

**Authors:** Tânia Carlice-dos-Reis, Jaime Viana, Fabiano Cordeiro Moreira, Greice de Lemos Cardoso, João Guerreiro, Sidney Santos, Ândrea Ribeiro-dos-Santos

**Affiliations:** 1 Laboratory of Human and Medical Genetics, Institute of Biological Sciences, Federal University of Pará, Belém, PA, Brazil; 2 Federal Rural University of the Amazon, Capanema Campus, PA, Brazil; 3 Research Center of Oncology, Federal University of Pará, Belém, PA, Brazil; Odense University Hospital, DENMARK

## Abstract

Mutations in the *HBB* gene are responsible for several serious hemoglobinopathies, such as sickle cell anemia and β-thalassemia. Sickle cell anemia is one of the most common monogenic diseases worldwide. Due to its prevalence, diverse strategies have been developed for a better understanding of its molecular mechanisms. *In silico* analysis has been increasingly used to investigate the genotype-phenotype relationship of many diseases, and the sequences of healthy individuals deposited in the 1,000 Genomes database appear to be an excellent tool for such analysis. The objective of this study is to analyze the variations in the *HBB* gene in the 1,000 Genomes database, to describe the mutation frequencies in the different population groups, and to investigate the pattern of pathogenicity. The computational tool SNPEFF was used to align the data from 2,504 samples of the 1,000 Genomes database with the HG19 genome reference. The pathogenicity of each amino acid change was investigated using the databases CLINVAR, dbSNP and HbVar and five different predictors. Twenty different mutations were found in 209 healthy individuals. The African group had the highest number of individuals with mutations, and the European group had the lowest number. Thus, it is concluded that approximately 8.3% of phenotypically healthy individuals from the 1,000 Genomes database have some mutation in the *HBB* gene. The frequency of mutated genes was estimated at 0.042, so that the expected frequency of being homozygous or compound heterozygous for these variants in the next generation is approximately 0.002. In total, 193 subjects had a non-synonymous mutation, which 186 (7.4%) have a deleterious mutation. Considering that the 1,000 Genomes database is representative of the world’s population, it can be estimated that fourteen out of every 10,000 individuals in the world will have a hemoglobinopathy in the next generation.

## 1. Introduction

Understanding the relationship between phenotype and genotype in the clinical setting is one of the main objectives of traditional research [1]. However, studies on a large number of mutations are problematic, primarily due to the experimental analyses. In contrast, *in silico* analysis is faster and easier to execute, yields more results, and costs less, thus making it more efficient. This type of analysis is based on alterations in the sequences of nucleotides and/or amino acids and their comparison with the native sequence to correlate the effect of these alterations on the phenotype of the individual [1,2,3,4].

Mutations in the *HBB* gene, which is located on chromosome 11 p15.5 [5], are responsible for several serious hemoglobinopathies, such as sickle cell anemia and β-thalassemia. Hemoglobinopathies are a set of hereditary diseases caused by the abnormal structure or insufficient production of hemoglobin. Sickle cell anemia and β-thalassemia can lead to serious anemia and other life threatening conditions [6]. Sickle cell anemia is one of the most common monogenic diseases worldwide. It is estimated that 312,000 people are born with sickle cell anemia every year, and the majority of these individuals are native to Sub-Saharan Africa [7]. Thus, it is important for the public healthcare system to detect heterozygous carriers of hemoglobinopathies, as they can produce homozygous and double heterozygous individuals with serious clinical conditions [8].

The 1,000 Genomes Project is an international consortium organized with the objective of sequencing a large number of individual genomes representative of the world’s population. The consortium has the objective of better characterizing the sequence variation of the human genome and enabling the investigation of the relationship between genotype and phenotype. Thus, the 1,000 Genomes Project enables a more precise study of variants in genome-wide association studies (GWAS) and the best localization of variants associated with diseases in different population groups [9].

The objective of this study is to track variations in the β-globin gene (*HBB*); to describe the frequencies of mutations in different population groups using the 1,000 Genomes databank, which provides a comprehensive resource of human genetic variation [9] relative to the HG19 reference genome [10]; and to investigate the pattern of resulting pathogenicity.

## 2. Methodology

To perform this study, data from 2,504 samples deposited in the 1,000 Genomes database were used; these open-access sequences were aligned with the HG19 reference genome using the SNPEFF tool [11]. This program provides and records the effects both of genetic variations as well as amino acid alterations. The resulting data were visualized in the Integrative Genomics Viewer (IGV) [12], a high-performance visualization tool for the interactive exploration of genomic datasets. The mutations were tracked at the nucleotide and amino acid levels, and the population frequencies with which these mutations occur, the type of mutation, and the respective positions were recorded.

**Fig 1:**
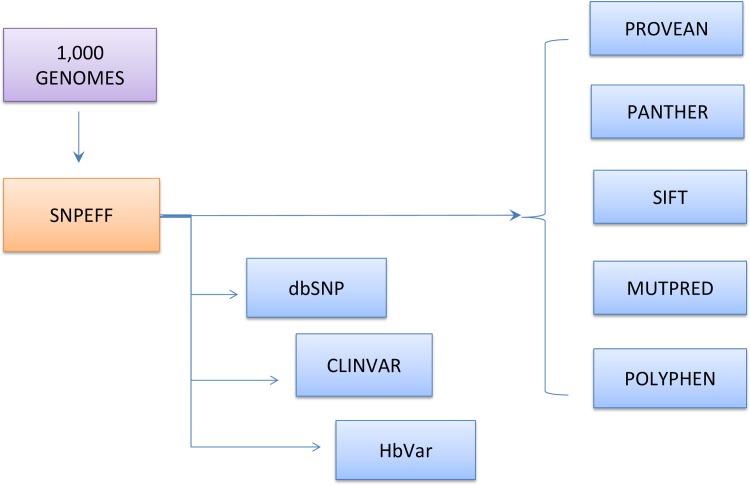
Alignment of the 1000 Genomes and HG19 sequences of *HBB* using the SNPEFF tool; predictors and BD used for the investigation of pathogenic mutations.

To investigate pathogenicity these mutations, five different prediction tools, including POLYPHEN [13], SIFT [14], PROVEAN [15], PANTHER [16], and E MUTPRED [17], and three databanks, including CLINVAR [18], dbSNP [19] and HbVar [20], were used, as shown in Fig 1.

Each predictor uses distinct characteristics to determine the effect of the mutations in relation to the information obtained regarding the structure and function of the protein. It is important to highlight that the results of all predictors provide additional evidence of pathogenicity; thus, five predictors were analyzed to improve accuracy. The determination of the pathogenicity of each mutation is based on four pieces of evidence: (i) CLINVAR, (ii) dbSNP, (iii) HbVar, and (iv) predictors.

Tables 1, 2 and 3 present the following results of the alignment of sequences from 2,504 samples: (1) the positions in the genome; (2) the identification of the single nucleotide polymorphism (SNP) of each mutation; (3) the types of mutations; (4) the mutations observed at the nucleotide level; (5) the respective consequences at the amino acid level; (6) the population frequency of each mutation; and (7) the pathogenicity investigated for each mutation.

**Table 1:** Position and SNP ID of the mutations observed at the nucleotide level, the respective consequences at the amino acid level, the types of mutations, and the number of individuals.

Position	SNP ID	Nucleotide change	AA alteration	Type of mutation	N° Individuals	Ref.
5246840	rs36020563	G/A	His144His	Synonymous	1	[21]
5246870	rs113082294	C/G	Val134Val	Synonymous	9	[22]
5246883	rs111645889	G/A	Ala130Val	Missense	1	[23]
5246890	rs33971634	G/A	Gln128	Stop gained	1	[24]
5246908	rs33946267	C/G	Glu122Gln	Missense	3	[25]
5246947	rs33958637	T/G	Asn109His	Missense	1	[26]
5246948	rs193922562	G/A	Gly108Gly	Synonymous	1	[27]
5247876	rs145669504	G/T	Leu82Leu	Synonymous	5	[28]
5247992–5247996	rs281864900	CAAAG/C	Phe42fs	Frameshift	5	[29]
5248004	rs11549407	G/A	Gln40	Stop gained	1	[30]
5248029	rs1135071	C/A	Arg31Ser	Splice region and missense	1	[31]
5248030	rs33943001	C/G	#	Splice acceptor and intron variant	1	[32]
5248159	rs33971440	C/T	#	Splice donor and intron variant	1	[33]
5248162	rs35578002	G/T	Glu30Gly	Splice region and synonymous variant	1	[34]
5248173	rs33950507	C/T	Glu27Lys	Missense	14	[35]
5248200	rs33986703	T/A	Lys18	Stop gained	6	[36]
5248205	rs63750783	C/T	Trp16	Stop gained	2	[37]
5248232	rs334	T/A	Glu7Val	Missense	137	[38]
5248233	rs33930165	C/T	Glu7Lys	Missense	17	[39]
5248236	rs33912272	G/A	Pro6Ser	Missense	1	[40]

#—Intronic variant mutations

**Table 2:** SNP ID, nucleotide and Amino Acid changes, number of individuals and population frequency of each mutation.

SNP ID	Nucleotide change	Amino Acid change	Total individuals	N°/ Freq AFR	N°/ Freq AMR	N°/ Freq EAS	N°/Freq EUR	N°/ Freq SAS	Total Allele Frequency
rs36020563	G/A	His144His	1	1 (0.0008)	0	0	0	0	0.00019
rs113082294	C/G	Val134Val	9	0	2 (0.0029)	0	7 (0.007)	0	0.00179
rs111645889	G/A	Ala130Val	1	1 (0.0008)	0	0	0	0	0.00019
rs33971634	G/A	Gln128	1	0	1 (0.0014)	0	0	0	0.00019
rs33946267	C/G	Glu122Gln	3	0	0	0	0	3 (0.0031)	0.00059
rs33958637	T/G	Asn109His	1	0	0	1 (0.001)	0	0	0.00019
rs193922562	G/A	Gly108Gly	1	1 (0.0008)	0	0	0	0	0.00019
rs145669504	G/T	Leu82Leu	5	0	0	5 (0.005)	0	0	0.00099
rs281864900	CAAAG/C	Phe42fs	5	0	0	5 (0.005)	0	0	0.00099
rs11549407	G/A	Gln40	1	0	1 (0.0014)	0	0	0	0.00019
rs1135071	C/A	Arg31Ser	1	0	0	0	1 (0.001)	0	0.00019
rs33943001	C/G	#	1	0	0	0	0	1 (0.001)	0.00019
rs33971440	C/T	#	1	0	1 (0.0014)	0	0	0	0.00019
rs35578002	G/T	Glu30Gly	1	1 (0.0008)	0	0	0	0	0.00019
rs33950507	C/T	Glu27Lys	14	0	0	8 (0.0079)	0	6 (0.0061)	0.00279
rs33986703	T/A	Lys18	6	0	0	6 (0.006)	0	0	0.00119
rs63750783	C/T	Trp16	2	0	0	0	0	2 (0.002)	0.00039
rs334	T/A	Glu7Val	137	132 (0.0072)	5 (0.0998)	0	0	0	0.02735
rs33930165	C/T	Glu7Lys	17	17 (0.0129)	0	0	0	0	0.00339
rs33912272	G/A	Pro6Ser	1	0	0	0	1 (0.001)	0	0.00019

AFR: African.; AMR: American; EAS: Eastern Asian; EUR: European; SAS: Southern Asian.

**Table 3:** SNP ID; nucleotide alteration; amino acid alteration; total number of individuals; list of the results from CLINVAR, dbSNP, HbVar, POLYPHEN, PROVEAN, SIFT, PANTHER, and MUTPRED; and final analysis of pathogenicity.

SNP ID	Nucleotide change	Amino acid change	Total individuals	CLINVAR	dbSNP (NCBI)	HbVar	POLYPHEN	PROVEAN	SIFT	PANTHER	MUTPRED	Conclusion pathogenicity
rs11164588	G/A	Ala130Val	1	Other	Other	Benign	Benign	Damaging	Damaging	Damaging	Damaging	Conflict
rs33971634	G/A	Gln128	1	Damaging	Other	Damaging	*	Damaging	*	*	*	Damaging
rs33946267	C/G	Glu122Gln	3	Damaging	Damaging	Benign	Benign	Benign	Damaging	Benign	Damaging	Conflict
rs33958637	T/G	Asn109His	1	Other	*	Benign	Probably damaging	Damaging	Damaging	Benign	Damaging	Conflict
rs281864900	CAAAG/C	Phe42fs	5	Damaging	Damaging	Damaging	*	Damaging	*	*	*	Damaging
rs11549407	G/A	Gln40	1	Damaging	Damaging	Damaging	*	*	*	*	*	Damaging
rs1135071	C/A	Arg31Ser	1	Damaging	Damaging	Benign	Probably damaging	Damaging	Damaging	Damaging	Damaging	Damaging
rs33943001	C/G	#	1	Damaging	Damaging	Damaging	*	*	*	*	*	Damaging
rs33971440	C/T	#	1	Damaging	Damaging	Damaging	*	*	*	*	*	Damaging
rs35578002	G/T	Glu30Gly	1	*	*	Damaging	Benign	Benign	Benign	Benign	Benign	Conflict
rs33950507	C/T	Glu27Lys	14	Damaging	Damaging	Damaging	Benign	Damaging	Damaging	Damaging	Damaging	Damaging
rs33986703	T/A	Lys18	6	Damaging	Damaging	Damaging	*	Damaging	*	*	*	Damaging
rs63750783	C/T	Trp16	2	Damaging	Damaging	Damaging	*	Damaging	*	*	*	Damaging
rs334	T/A	Glu7Val	137	Damaging	Damaging	Damaging	Benign	Damaging	Damaging	*	Damaging	Damaging
rs33930165	C/T	Glu7Lys	17	Damaging	Damaging	Damaging	Benign	Damaging	Damaging	*	Damaging	Damaging
rs33912272	G/A	Pro6Ser	1	Other	Other	Benign	Benign	Benign	Benign	*	Damaging	Conflict

* Could not be evaluated

# Intronic variant mutations

## 3. Results

A total of 20 different mutations were identified in 209 individuals (8.34%) in the coding region of the *HBB* gene. The variants observed were classified as follows: (i) four synonymous mutations; (ii) seven missense mutations; (iii) four stop-gain mutations; (iv) one frameshift mutation; (v) one splice region and missense variant; (vi) one splice region and synonymous variant; (vii) one splice acceptor and intron variant; and (viii) one splice donor and intron variant. Missense mutations were the most frequently encountered, affecting 174 (83.2%) individuals, as shown in Table 1. All observed mutations were heterozygous and already had SNP IDs.

The mutations with the highest allelic frequencies were as follows: (i) rs334 had total frequency of 0.0274 (African and American populations); (ii) rs33930165 had a frequency of 0.0034 (only in the African population); and (iii) rs33950507 had a frequency of 0.0028 (Eastern and Southern Asian populations), as shown in Table 2.

Synonymous mutations were encountered in 16 (7.6%) samples and were excluded from the investigation of pathogenicity performed by the database predictors because they do not alter the amino acid sequence.

Thus, the pathogenicity of missense, stop-gain, frameshift, splice region (both acceptor and donors), synonymous and intron mutations were tracked using the dbSNP, CLINVAR and HbVar databases, as well as five *in silico* predictors (POLYPHEN, SIFT, PROVEAN, PANTHER and MUTPRED). The results showed 11 pathogenic mutations of *HBB* (Table 3). In addition, five mutations—(1) rs111645889, (2) rs33946267, (3) rs33958637 (4) rs35578002 and (5) rs33912272—presented conflicting results between predictors and databases.

## 4. Discussion

Mutations in the *HBB* gene are distributed unevenly among the different population groups. The African population was the most affected, with 73.2% of individuals having mutations in this gene, while the European population was least affected, with 4.3% of individuals having such mutations.

The three mutations with the greatest frequency were (1) rs334 (AFR and AMR); (2) rs33930165 (AFR); and (3) rs33950507 (EAS and SAS). The rs334 mutation is responsible for hemoglobin S, known as *HbS*, which causes sickle cell anemia. The rs33930165 mutation is responsible for hemoglobin C, or *HbC* [41], which is more frequent in the African population [42,43]. In addition, the rs3395057 mutation is responsible for hemoglobin E, or *HbE* [41], which is involved in β-thalassemia described in Asian populations [44].

The available data show that variants rs33986703, rs63750783, and rs281864900 are responsible for β-thalassemia and are described in Asian populations [45,46,39]. Variants rs11549407 and rs33971634 are also β-thalassemia mutations but are common in European populations [47,24]; rs33971440 and rs35578002 are commonly found in populations of the Mediterranean region [48,49,34].

Although the *HBB* gene is well studied, there are some mutations in this gene that are not well known and poorly described in the literature. This is the case of the variants rs111645889, rs33958637, rs1135071, rs33943001 and rs33912272, for which no scientific papers were found discussing their epidemiology.

CLINVAR [18] is one of the most widely used databases in clinical and pathological analyses related to mutations. However, not all mutations of the *HBB* gene (rs35578002) are registered in this database, and conflicting results have been observed when comparing predictors with the CLINVAR, dbSNP and HbVar databases to estimate the pathogenicity of each mutation, or more specifically, the clinical significance of mutations rs111645889, rs33946267, rs33958637, rs35578002 and rs33912272.

It is important to emphasize that all samples deposited in the 1,000 Genomes Project, an international consortium aimed at producing a public catalog of human genetic variability, belong to individuals without clinical manifestations of any disease.

The SNP rs35578002 is not available in CLINVAR and has no information on clinical significance in the dbSNP database. Predictors consider this variant as benign, but the HbVar database classifies it as a damaging mutation. This variant is the β-thalassemia mutation Cd29 (C> T), which in homozygosis causes hemolytic anemia and ineffective erythropoiesis [34]. This mutation was described in Mediterranean populations. One possible explanation for the inconsistent information about the clinical significance of this variant is that it is a synonymous mutation in the splice region that is critical for RNA processing, causing thalassemia as described in HbVar. Also noteworthy is the mutation rs33946267. According to the literature, this mutation leads to the formation of Hb D-Punjab. This mutation is generally asymptomatic but may occasionally cause moderate hemolytic anemia, similar to the manifestations of sickle cell anemia when associated with other hemoglobin variants, such as HbS or β-thalassemia mutations. Its initial distribution suggests that it is more prevalent in the central region of Asia, but due to migration, it can be found in several other regions [50].

According to the results, 8.3% of the phenotypically healthy individuals of the 1,000 Genomes database have a mutation in the *HBB* gene in heterozygosis. This means that eighty out of 1,000 individuals have a mutant allele in the gene. The frequency of mutated genes was estimated at 0.042, so that the expected frequency of being homozygous or compound heterozygous for these variants in the next generation is approximately 0.002. In total, 193 subjects had a non-synonymous mutation, meaning that approximately 7.7% had a change that affects the sequence of amino acids. Of these, 186 (7.4%) have a deleterious mutation based on available data on the clinical significance of these mutations (Table 3). Considering that the 1,000 Genomes database is representative of the world’s population, it can be estimated that fourteen out of every 10,000 individuals in the world will have a hemoglobinopathy in the next generation.

Independently, new studies are needed to validate the clinical consequences of the mutations with undefined pathogenicity. Considering the absence of physiopathological knowledge relative to the newly identified mutations, the use of *in silico* predictors (in an orderly and criteria-based manner) emerges as a possible tool to aid in decision-making with respect to diagnostic, preventative, and treatment measures.

## References

[1] Singh PK, Mistry KN. A computational approach to determine susceptibility to cancer by evaluating the deleterious effect of nsSNP in XRCC1 gene on binding interaction of XRCC1 protein with ligase III. Gene. 2016; 576: 141–149. 10.1016/j.gene.2015.09.084 26449312

[2] Lettre G. The search for genetic modifiers of disease severity in the β-hemoglobinopathies. Cold Spring Harb. Perspect. Med. 2012, 2(10):1–12. 10.1101/cshperspect.a015032 PMC3475403 23028136

[3] Steinberg MH, Sebastiani P. Genetic modifiers of sickle cell disease. Am. J. Hematol. 2012; 87(8): 795–803. 10.1002/ajh.23232 22641398 PMC4562292

[4] Steinberg MH. Genetic etiologies for phenotypic diversity in sickle cell anemia. Sci World J 2009; 9:46–67 10.1100/tsw.2009.10 PMC5823205 19151898

[5] Onda M, AkaishI J, Asaka S, Okamoto J, Miyamoto S, Mizutani K, et al Decreased expression of haemoglobin beta (HBB) gene in anaplastic thyroid cancer and recovory of its expression inhibits cell growth. British Journal of Cancer. 2005; 92: 2216–2224. 10.1038/sj.bjc.6602634 15956966 PMC2361827

[6] Li M, Suzuki K, Qu J, Saini P, Dubova I, Yi F, et al Efficient correction of hemoglobinopathy-causing mutationsby homologous recombination in integration-free patient iPSCs. Cell Research. 2011; 21: 1740–1744. 10.1038/cr.2011.186 22105484 PMC3357996

[7] Saraf SL, Molokie RE, Nouraire M, Sable CA, Jones LL, Ensing GJ, et al Differences in the clinical and genotypic presentation of sickle cell disease around the world. Paediatr Respir Rev 2014; 15: 4–12. 10.1016/j.prrv.2013.11.003 24361300 PMC3944316

[8] Orlando GM, Naoum PC, Siqueira FAM, Bonini-Domingos CR. [Laboratory diagnosis of hemoglobinopathies in distinct populations]. Bras. Hematol. Hemoter. 2000; 22:111–121.

[9] The 1000 Genomes Project Consortium. A map of human genome variation from population- scale sequencing. Nature.2010; 467:1061–1073. 10.1038/nature09534 20981092 PMC3042601

[10] Miga KH, Newton Y, Jain M, Altemose N, Willard HF, Kent WJ. Centromere reference models for human chromosomes X and Y satellite arrays. Genome Res.2014; 24: 697–707. 10.1101/gr.159624.113 24501022 PMC3975068

[11] Cingolani P, Platts A, Wang LL, Coon M, Nguyen T, Wang L, et al A program for annotating and predicting the effects of single nucleotide polymorphisms, SnpEff: SNPs in the genome of Drosophila melanogaster strain w 1118; iso-2; iso-3. Landes Bioscience. 2012; 2: 1–13. 10.4161/fly.19695 PMC3679285 22728672

[12] Robinson JT, Thorvaldsdóttir H, Wincker W, Guttman M, Lander ES, Getz G, et al Integrative genomics viewer. Nature biotechnology. 2011; 29: 24–26. 10.1038/nbt.1754 21221095 PMC3346182

[13] Adzhubei VA, Schmidt S, Peshkin L, Ramensky VE, Gerasimova A, Bork P, et al A method and server for predicting damaging missense mutations. Nature methods. 2010; 7: 248–249. 10.1038/nmeth0410-248 20354512 PMC2855889

[14] Kumar P, Henikoff S, Ng PC. Predicting the effects of coding non-synonymous variants on protein function using the SIFT algorithm. Nature protocols. 2009; 4: 1073–1082. 10.1038/nprot.2009.86 19561590

[15] Choi Y, Sims GE, Murphy S, Miller JR, Chan AP. Predicting the Functional Effect of Amino Acid Substitutions and Indels. Plos One. 2012; 7: 1–13. 10.1371/journal.pone.0046688 PMC3466303 23056405

[16] Mi H, Muruganujan A, Thomas PD. PANTHER in 2013: modeling the evolution of gene function, and other gene attributes, in the context of phylogenetic trees. Nucleic Acids Research. 2013; 41: 377–386. 10.1093/nar/gks1118 PMC3531194 23193289

[17] Li B, Krishman VG, Mort ME, Xin F, Kamati KK, Cooper DN, et al Automated inference of molecular mechanisms of disease from amino acid substitutions. Bioinformatics. 2009; 25: 20744–2750. 10.1093/bioinformatics/btp528 PMC3140805 19734154

[18] Landrum MJ, Lee JM, Riley GR, Jang W, Rubinstein WS, Church MD, et al ClinVar: public archive of relationships among sequence variation and human phenotype. Nucleic Acids Research.2014; 42: 980–985. 10.1093/nar/gkt1113 PMC3965032 24234437

[19] Kitts A, Sherry S. Chapter 5 The Single Nucleotide Polymorphism Database (dbSNP) of Nucleotide Sequence Variation. The NCBI Handbook [Internet].2002. Disponível em < http://www.ncbi.nlm.nih.gov/books/NBK21088/>.

[20] Hardison RC, Chui DHK, Giardine B, Riemer C, Patrinos GP, Anagnou N, Miller W, Wajcman H. Hb Var.A Relational Database of Human Hemoglobin Variants and Thalassemia Mutation at the globin Gene Server. 2002; 19: 225–233. 10.1002/humu.10044 11857738

[21] CLlNVAR Database, https://www.ncbi.nlm.nih.gov/clinvar/variation/36329/; Last evaluated: Aug 18, 2011 [accessed 18.01.17].

[22] Pianezze G, Toniolo M, Masieri MT, Dolcini B, Ravani A. Hb Belluno [β111(G13)Val→Gly;β133(H11)Val→Val (HBB: c.335T > G;402G > C)]: Incidental Detection of a New Clinically Silent β Chain Variant During Hb A1c Determination by High Performance Liquid. Hemoglobin. 2016; 40: 143–149. 10.3109/03630269.2016.1150292 27032675

[23] Merault G, Keclard L, Garin J, Poyart C, Blouquit Y, Arous N, Galacteros F, Feingold J, Rosa J. Hemoglobin La Desirade alpha A2 beta 2 129 (H7) Ala—-Val: a new unstable hemoglobin. Hemoglobin. 1986; 10: 593–605. 3557994 10.3109/03630268609036564

[24] Hall GW, Franklin IM, Thein SL. A novel mutation(nonsense beta 127) in exon 3 of beta globin gene produces a variable thalassaemic phenotype. British journal of Haematology. 1991; 79: 342–344. 1958498 10.1111/j.1365-2141.1991.tb04548.x

[25] Schneider RG, Ueda S, Alperin JB, Levin WC, Jones RT, Brimhall B. Hemoglobin D Los Angeles in two Caucasian families: hemoglobin SD disease and hemoglobin D thalassemia. Blood. 1968; 32: 250–259. 5672850

[26] Imamura T, Fujita S, Ohta Y, Hanada M, Yanase T. Hemoglobin Yoshizuka (G10(108)beta asparagine—aspartic acid): a new variant with a reduced oxygen affinity from a Japanese family. The Journal of Clinical Investigation. 1969; 48: 2341–2348. 10.1172/JCI106200 5355345 PMC297491

[27] CLINVAR Database, https://www.ncbi.nlm.nih.gov/clinvar/variation/36322/; Last evaluated: Aug 18, 2011 [accessed 18.01.17].

[28] CLlNVARDatabase, https://www.ncbi.nlm.nih.gov/clinvar/variation/36305/; Last evaluated: Aug 18, 2011 [accessed 18.01.17].

[29] Kimura A, Matsunaga E, Takihara Y, Nakamura T, Takagi Y, Lin S, Lee H. Structural analysis of a beta-thalassemia gene found in Taiwan. The Journal of Biological Chemistry. 1983; 258: 2748–2749. 6826539

[30] Trecartin RF, Liebhaber SA, Chang JC, Lee KY, Kan YW, Furbetta M, Angius A, Cao A. Beta zero thalassemia in Sardinia is caused by a nonsense mutation. The American Society for Clinical Invetigation, inc. 1981; 68:1012–1017. 10.1172/JCI110323 PMC370888 6457059

[31] Baur EW, Motulsky AG. Hemoglobin tacoma—a beta-chain variant associated with increased hb A2. Humangenetik. 1965; 1: 621–634. 5869485

[32] Deidda G, Novelletto A, Hafez M, al-Tonbary Y, Felicetti L, Terrenato L, Colombo B. A new beta-thalassemia mutation produced by a single nucleotide substitution in the conserved dinucleotide sequence of the IVS-I consensus acceptor site (AG—-AA). Hemoglobin. 1990; 14: 431–440. 2283297 10.3109/03630269009032003

[33] Orkin SH, Kazazian HH Jr, Antonarakis SE, Goff SC, Boehm CD, Sexton JP, Waber PG, Giardina PJ. Linkage of beta-thalassaemia mutations and beta-globin gene polymorphisms with DNA polymorphisms in human beta-globin gene cluster. Nature. 1982; 296: 627–631. 6280057 10.1038/296627a0

[34] Chehab FF, Der Kaloustian V, Khouri FP, Deeb SS, Kan YW. The molecular basis of beta-thalassemia in Lebanon: application to prenatal diagnosis. Blood. 1987; 69: 1141–1145. 3828533

[35] Kazazian HH Jr, Waber PG, Boehm CD, Lee JI, Antonarakis SE, Fairbanks VF. Hemoglobin E in Europeans: further evidence for multiple origins of the beta E-globin gene. Am J Hum Genet. 1984; 36: 212–217. 6198908 PMC1684388

[36] Chang JC, Kan YW. Beta 0 thalassemia, a nonsense mutation in man. Proc. Natl. Acad. Sci. 1979; 76: 2886–2889. 88735 10.1073/pnas.76.6.2886 PMC383714

[37] Aulehla-Scholz C, Basaran S, Agaoglu L, Arcasoy A, Holzgreve W, Miny P, Ridolfi F, Horst J. Molecular basis of beta-thalassemia in Turkey: detection of rare mutations by direct sequencing. Human Genet. 1990; 84: 195–197. 2298457 10.1007/BF00208941

[38] Blackwell RQ, Oemijati S, Pribadi W, Weng MI, Liu CS. Hemoglobin G Makassar: beta-6 Glu leads to Ala. Biochimica et Biophysica Acta. 1970; 214: 396–401. 5509617

[39] Harano T, Harano K, Ueda S, Shibata S, Imai K, Seki M. Hemoglobin Machida [beta 6 (A3) Glu replaced by Gln], a new abnormal hemoglobin discovered in a Japanese family: structure, function and biosynthesis. Hemoglobin. 1982; 6: 531–535. 6129204 10.3109/03630268209083766

[40] Langdown JV, Williamson D, Beresford CH, Gibb I, Taylor R, Deacon-Smith R. A new beta chain variant, Hb Tyne [beta 5(A2)Pro—>Ser. Hemoglobin. 1994; 18: 333–336. 7852088 10.3109/03630269408996199

[41] Lelliott PM, McMorran BJ, Foote SJ, Burgio G. The influence of host genetics on erythrocytes and malaria infection: is there therapeutic potential? Malaria Journal.2015; 14: 1–15. 26215182 10.1186/s12936-015-0809-x PMC4517643

[42] Carter TE, Fricken MV, Romain JR, Memmon G, Victor YS, Shick L, Okech BA, Mulligan CJ. Detection of Sickle Cell Hemoglobin in Haiti by Genotyping and Hemoglobin Solubility Tests. Am J. Trop. Med. Hyg. 2014; 91: 406–411. 10.4269/ajtmh.13-0572 24957539 PMC4125270

[43] Gansah A, Rockett KA, Clark TG, Wilson MD, Koram KA, Oduro AR, Amenga-Etego L, Anyorigiya T, Hodgson A, Milligan P, Rogers WO, Kwiatkowski DP. Haplotype analyses of haemoglobin C and haemoglobin S and the dynamics of evolution response to malaria in Kassena-Nankana district of Ghana. PLos ONE. 2012; 7: e34565 10.1371/journal.pone.0034565 22506028 PMC3323552

[44] Sultana GNN, Begum R, Akhter H, Shamim Z, Rahim MA, Chaubey G. The complete Spectrum of beta (β) thalassemia mutations in Bangladeshi population. 2016; 3 (1): 1–6

[45] Chang JC, Kan YW. B^0^ thalassemia, a nonsense mitation in man. Proc. Natl. Acad. Sci. 1979; 76: 2886–2889. 88735 10.1073/pnas.76.6.2886 PMC383714

[46] Kulkarni GD, Kulkarni SS, Kadakol GS, Kulkarni BB, Kyamangoudar PH, Lakkakula BVKS, Thangaraj K, Shepur TA, Kulkarni ML, Gai PB. Molecular basis of β -thalassemia in Karnataka, India. Genetic Testing and Molecular Biomarkers. 2012; 16: 138–141. 10.1089/gtmb.2011.0035 21978377

[47] Danjou F, Zoledziewska M, Sidore C, Steri M, Busonero F, Maschio A, Mulas A, Perseu L, Barella S, Porcu E, Pistis G, Pitzalis M, Pala M, Menzel S, Metrustry S, Spector TD, Leoni L, Angius A, Uda M, Moi P, Thein SL, Galanello R, Gonçalo R Abecasis GR, Schlessinger D, Sanna S, Cucca F. Genome-wide association analyses based on whole-genome sequencing in Sardinia provide insights into regulation of hemoglobin levels. Nature genetics. 2015; 47: 1264–1271. 10.1038/ng.3307 26366553 PMC4627580

[48] Sirdah MM, Sievertsen J, Al-Yazji MS, Tarazi IS, Al-Haddad RM, Horstmann RD, Timmann C. The spectrum of β-thalassemia in Gaza strip, Palestine. Blood Cells, Molecules and Diseases. 2013; 50: 247–251. 10.1016/j.bcmd.2012.12.004 23321370

[49] Chassanidis C, Boutou E, Voskaridou E, Balassopoulou A. Development of a high-resolution melting approach for scanning beta globin gene point mutations in the Greek and other Mediterranean populations.PLos ONE. 2016; 11: e0157393 10.1371/journal.pone.0157393 27351925 PMC4924799

[50] Torres LS, Okumura JV, Silva DGH, Bonini-Domingos CR. Hemoglobin D-Punjab: origin, distribution and laboratiry diagnosis. Rev Bras Hematol Hemoter. 2015; 37: 120–126. 10.1016/j.bjhh.2015.02.007 25818823 PMC4382585

